# Low-Cost Resin 3-D Printing for Rapid Prototyping of Microdevices: Opportunities for Supporting Aquatic Germplasm Repositories

**DOI:** 10.3390/fishes7010049

**Published:** 2022-02-15

**Authors:** Nikolas C. Zuchowicz, Jorge A. Belgodere, Yue Liu, Ignatius Semmes, William Todd Monroe, Terrence R. Tiersch

**Affiliations:** 1 Aquatic Germplasm and Genetic Resources Center, School of Renewable Natural Resources, Louisiana State University Agricultural Center, Baton Rouge, LA 70820, USA; 2 Department of Biological and Agricultural Engineering, Louisiana State University Agricultural Center, Baton Rouge, LA 70803, USA; 3 Department of Biological and Agricultural Engineering, Louisiana State University, Baton Rouge, LA 70803, USA

**Keywords:** sperm, 3-D printing, resin, stereolithography, microfabrication, millifluidic

## Abstract

Germplasm repositories can benefit sustainable aquaculture by supporting genetic improvement, assisted reproduction, and management of valuable genetic resources. Lack of reliable quality management tools has impeded repository development in the past several decades. Microfabricated open-hardware devices have emerged as a new approach to assist repository development by providing standardized quality assessment capabilities to enable routine quality control. However, prototyping of microfabricated devices (microdevices) traditionally relies on photolithography techniques that are costly, time intensive, and accessible only through specialized engineering laboratories. Although resin 3-D printing has been introduced into the microfabrication domain, existing publications focus on customized or high-cost (>thousands of USD) printers. The goal of this report was to identify and call attention to the emerging opportunities to support innovation in microfabrication by use of low-cost (<USD 350) resin 3-D printing for rapid prototyping. We demonstrate that low-cost mask-based stereolithography (MSLA) 3-D printers with straightforward modifications can provide fabrication quality that approaches traditional photolithography techniques. For example, reliable feature sizes of 20 μm with dimensional discrepancy of <4% for lateral dimensions and <5% for vertical dimensions were fabricated with a consumer-level MSLA printers. In addition, alterations made to pre-processing, post-processing, and printer configuration steps improved print quality as demonstrated in objects with sharper edges and smoother surfaces. The prototyping time and cost of resin 3-D printing (3 h with USD 0.5/prototype) were considerably lower than those of traditional photolithography (5 d with USD 80/prototype). With the rapid advance of consumer-grade printers, resin 3-D printing can revolutionize rapid prototyping approaches for microdevices in the near future, facilitating participation in interdisciplinary development of innovative hardware to support germplasm repository development for aquatic species.

## 1. Introduction

There is increasing demand for development of germplasm repositories through cryopreservation for aquatic species to support aquaculture [1], biomedical research [2], and conservation [3]. Germplasm cryopreservation can support sustainable aquaculture as a tool for management of valuable genetic resources, such as genetic lines with disease resistance, environmental adaptation, and faster and larger growth [4,5]. Quality management through quality control (QC), quality assurance (QA), and quality evaluation (QE) is indispensable in development of robust repositories to ensure long-term genetic resource storage and management. However, the lack of reliable hardware for quality management has substantially impeded repository development for aquatic species for decades [6]. For example, standard hemocytometers are commonly used in sperm counting due to their low cost; however, the chamber height of 100 μm was not designed for counting of fish sperm (<10 μm for most aquaculture species) and can lead to erroneous counts [7]. Commercial solutions for sperm counting are costly, uncustomizable, and fail to address the diversity of aquatic species (e.g., sperm size differences between fish and amphibians can be 10-fold). In recent years, microfabrication (“Lab-on-a-chip”) technologies have been applied for the creation of novel devices (microdevices) to enable quality assessment of samples to facilitate routine quality control programs. For example, microdevices have been developed for evaluation of sperm motility [8,9] and concentration [7] of aquatic species. These devices can be standardized with community-level efforts and customized to accommodate certain species. In addition, microdevices can facilitate research and applied practices in aquatic species, such as disease diagnosis [10] and detection of water contamination [11].

Prototyping of microfabricated devices has traditionally relied on costly and time-intensive techniques, such as photolithography, micro-milling, and hot embossing [12]. Industrial and commercial three-dimensional (3-D) resin printers have been recently introduced to microfabrication with photo-curable resins [13], which offers possibilities for prototyping with less time and expertise compared with traditional techniques [14]. Until recently, laser and digital light processing (DLP) stereo lithography (SLA) have been the dominant approaches in resin 3-D printing. Laser-SLA printers use ultraviolet (UV) lasers to cure resin with individual voxel (“volumetric element” or “3-dimensional pixel”) resolution, whereas DLP-SLA printers cure individual layers of resin with light patterns similar to DLP projectors [15]. Laser-SLA has been applied to rapid prototyping of microfluidic systems and world-to-chip interfaces with promising results [16]. Although laser and DLP resin printing have potential to reduce the costs of equipment, facilities, and staffing compared to traditional photolithography, previous publications addressing microfabrication with customized or higher-end resin technology used printers that cost thousands to hundreds of thousands of USD (Table 1), which poses a considerable barrier to entry for groups with limited budgets or experience such as fisheries and aquaculture researchers.

Open hardware and open fabrication are beginning to fuel a new movement toward distribution and participation in scientific equipment development among research communities [6,17]. Low-cost equipment is essential to bring open fabrication into the domain of open microfabrication. In the past three years, mask-based stereolithography (MSLA, or LED-LCD SLA) [18,19] resin 3-D printers have emerged on the consumer market. For example, MSLA has repurposed existing liquid crystal display (LCD) screens into masks to manipulate light patterns used to cure specific patterns into individual layers of resins [15]. The realization of this technology has opened the world of resin printing to the mass consumer market: for as low as USD 200, users can gain access to resin printers capable of a single-voxel resolution of 50 × 50 × 10 μm. It is unknown whether it is feasible to achieve microfabrication with these low-cost MSLA 3-D printers.

The goal of this report was to raise awareness of aquatic biologists to the emerging opportunities to support germplasm repository development by rapid prototyping of microdevices by use of low-cost (<USD 350) resin 3-D printers. Specific objectives were to: (1) introduce the possibility to fabricate micrometer-scale features with low-cost MSLA printers; (2) identify pre-processing, printer modification, and post-processing modifications that can enhance printing quality, and (3) compare the cost and time investment of MSLA printing to photolithography. We found that the features produced by low-cost MSLA printers could approach those produced by traditional photolithography techniques in quality and dimensions. The present work is not intended as technical engineering research to improve resin printing and microfabrication. Instead, our findings are intended to call attention to emerging opportunities for aquatic biologists to participate in interdisciplinary research, allowing entry into the fields of microfabrication and rapid prototyping to address major community-level challenges.

## 2. Materials and Methods

### 2.1. 3-D Printing

Standard T-channel geometries commonly used in microfluidic and millifluidic devices were used to assess microfabrication quality. The 3-D channels were created by use of computer-assisted design (CAD) in Fusion 360 software (Autodesk, San Rafael, CA, USA). The designed dimensions were: 210 μm for channel widths of the three branches, 630 μm for the width of the single main channel, and 20 μm for all channel depths.

A Phrozen Mini 4K (Phrozen Technology, Hsinchu City, Taiwan) MSLA printer was selected as a consumer-level MSLA printer with a cost of USD 349 (USD 299 at time of purchase). The CAD design was converted to STL format, imported to slicer software Lychee (mango3d.io, accessed 4 February 2022) to adjust print settings (Table 2), printed, and post-processed (i.e., cleaning and curing with additional UV exposure). Objects were printed with Siraya Tech Blu Clear v2 (Siraya Tech, San Gabriel, CA, USA) resin and a horizonal orientation.

### 2.2. Microfabrication by Traditional Photolithography

A master mold of the testing channels was patterned with SU-8 photoresist (MicroChem Corp., Newton, MA, USA) on a silicon wafer (Universitywafer.com, South Boston, MA, USA) with an existing protocol [9]. A 10:1 mixture (elastomer:curing agent) of Sylgard-184 (Dow Corning, Midland, MI, USA) polydimethylsiloxane (PDMS) was cast onto the master mold, degassed in a vacuum chamber, and cured in an oven at 65 °C for 3 h.

### 2.3. Evaluation of Fabrication Quality

The channel geometries fabricated by 3-D printing and photolithography were coated with platinum for 8 min using an Emitech K550X (Emitech Inc., Fall River, MA, USA) sputter coater to prepare surfaces. Objects were scanned by a Wyko Hi Res optical profiler (Bruker Nano, Inc., Tuscon, AZ, USA) with a step size of 30 μm. The surface profiles and surface roughness (root-mean-square height, RMS) were analyzed using MountainsMap software (v8, Digital Surf, Besançon, France). Surface roughness was obtained at two regions: a 0.4 × 1.6-mm rectangular region on the floor of the main channels and a 1.6 × 1.6-mm square region on the platform surfaces (adjacent to the channels). Scanning electron microscopy (SEM) imaging was performed using a JEOL JSM-6610LV SEM at the Shared Instrumentation Facility of Louisiana State University. SEM images were acquired at 15 kV, with a working distance ~45 mm and 60-× magnification.

### 2.4. Evaluation of Pre-Processing and Post-Processing

To perform printing pre-processing, dimensions were edited in Fusion 360 to produce desired design patterns that matched whole-number multiplicates of the size of the pixels (35 × 35 μm) of the LCD screen. This can improve fabrication accuracy by correction of the dimensions automatically assigned by the slicing software. For example, a designed dimension of 80 μm would be automatically rounded to 70 μm (2 × 35 μm) or 105 μm (3 × 35 μm) in the slicing software, resulting in undesired dimension alternations of the entire design. This default adjustment would not affect objects with features > mm but could greatly affect the geometrical accuracy of microfabrication. To evaluate pre-processing by dimension adjustment, two versions of a design with geometries for raised grid lines were created in the Fusion 360. For a control version, design geometries were not adjusted to match the pixel size, and 100-μm wide grid lines were used to form squares with a side length of 400 μm. The pre-processed version adjusted the width of grid lines to 105 μm (35 μm × 3) to form squares with side lengths of 385 μm (35 μm × 11). The designs were converted to STL format and sliced by Lychee (V3.3.8, Mango 3D, Bordeaux, France), which was used to visualize and inspect sliced dimensions displayed with unites of pixels. Objects were printed with the Phrozen Sonic Mini 4K resin printer using Siraya Tech Blu Clear v2 resin.

To evaluate effects of post-processing on printing quality, geometries with various shapes were created in Fusion 360, including letters, cylindrical pyramids, half-spheres (raised and sunken), and rectangular prisms (raised and sunken). These shapes can assist identification of fabrication quality [27,28] in various conditions that can interact with post-processing. One method followed protocols recommended by the manufacturer: printed pieces were rinsed with isopropyl alcohol (IPA) [29], dried with air, and UV-cured for 3 min. The improved method included additional steps: printed pieces were placed in IPA and cleaned in an ultrasonic bath for 3 min. The prints were removed and rinsed with fresh IPA and UV-cured for 3 min while submerged in fresh IPA.

### 2.5. Printer Modification for Surface Smoothness

Printer modifications were made to improve surface smoothness of the build plate by adhering (with super glue 15185 Gel that can be removed by soaking in acetone) a glass slide (5 cm × 7.5 cm) to a removable magnetic build surface (Wham Bam Systems, Hollywood, FL, USA) that attached to the build plate of the Phrozen Sonic Mini 4K printer. The channel design described above was printed with the first layer attached to the glass slide (instead of to the original steel build plate) with clear resin (Siraya Tech). Objects with and without these modifications were compared by visual assessment of light reflection in images taken with a camera and LED lights.

### 2.6. Comparison of Cost and Time Efficiencies

Comparisons of cost and time investment for microfabrication with traditional photolithography and resin 3-D printing were made by evaluation of routine operations within our facilities.

## 3. Results and Discussion

### 3.1. Fabrication Quality

**Figure 1.**
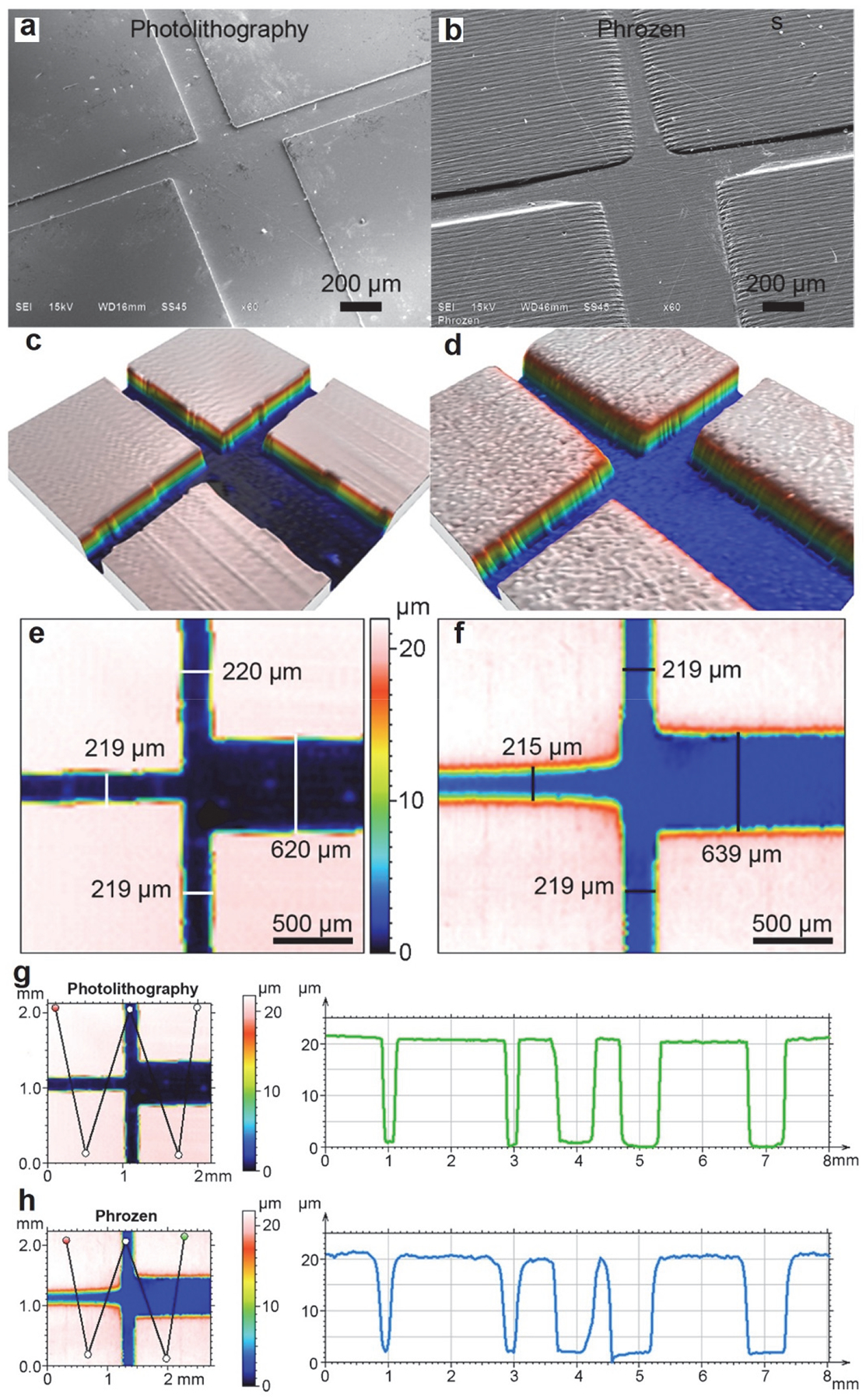
Evaluation of the fabrication quality for a “T”-channel using traditional photolithography and low-cost MSLA resin 3-D printing (Phrozen Mini 4K). The fabricated pieces were assessed with scanning electron microscopy (SEM) (**a**,**b**) and optical surface profiling (**c**–**f**). Profilometry was used to dimension the channel widths (**e**,**f**), with designed channel widths of 210 μm for three branched channels and 630 μm for a main channel. Profile lines were extracted to compare channel depth and feature resolution for multiple points across the chips (**g**,**h**).

The prints made by the low-cost MSLA (Phrozen Sonic 4K Mini) printer (Figure 1a) and by photolithography (Figure 1b) showed clearly defined channels. The comparability of fabrication quality was confirmed by profilometry, showing smooth surfaces with minimum defects produced by photolithography (Figure 1c) and MSLA 3-D printing (Figure 1d). The lateral channel widths of objects fabricated by MSLA (Figure 1f) were comparable to the photolithography printing (Figure 1e). The lateral (*x* and *y*-axes) discrepancies between nominal dimensions and actual prints were 2–5% for the photolithography print and 1–4% for the resin print. The line profile (Figure 1g,h) discrepancies for vertical channel heights (*z*-axis) were about 0–5% for photolithography and 0–10% for resin printing. Overall, the top and bottom surfaces produced by photolithography were better defined for flatness and squareness than those produced by resin printing. Rounding of features at the top and bottom corners was found in the MSLA prints. In addition, the surface roughness (RMS) of PDMS casts was 0.21 μm for the main channel and 0.17 μm for the platform, whereas the RMS of the resin print was 0.26 μm for the main channel and 0.79 for the platform.

Overall, the fabrication quality (i.e., dimensional accuracy and surface roughness) of microfluidic channels produced by the low-cost MSLA printer were comparable to those produced by traditional photolithography methods. Consumer-grade MSLA printers have been evolving at a rapid pace. For example, early-generation machines (e.g., the popular Anycubic Photon printers released three years ago) repurposed red-green-blue (RGB) LCDs with 2k (47.25-μm-pixel width) resolution that could produce fine details for features <1 mm [30]. Recent generations (e.g., Phrozen Sonic Mini 4K) have improved to monochrome-pixel LCDs with a 35-μm-pixel width. In comparison to the RGB type of LCDs, these ‘mono’ screens pass far more UV light with higher accuracy [31,32]. During the preparation of this manuscript, MSLA printers with higher resolutions (e.g., 8K resolutions) have begun to reach the market. We predict that the rapid pace of marketing and technological development of low-cost resin 3-D printing could resemble the evolution of the television or desktop computing markets, providing major opportunities for approaching reliable microfabrication in the near future.

### 3.2. Pre-Processing

**Figure 2.**
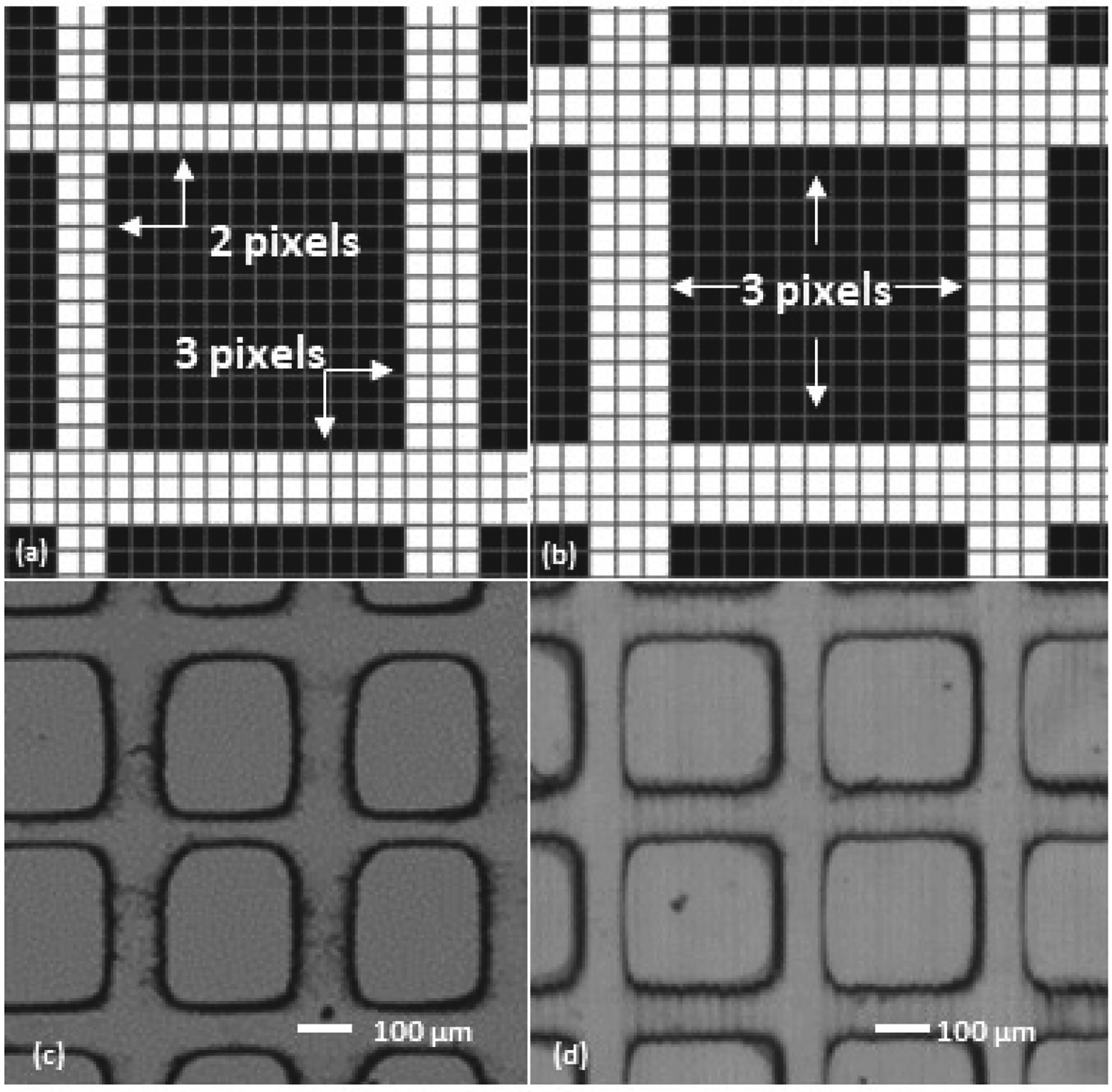
Evaluation of the effects of dimensional adjustment for pre-processing on fabrication quality by resin 3-D printing of designed grid lines (100 μm wide) and squares (400 × 400 μm). In UVtools software, dimensional alternations in the slicing process can be visualized. Without adjustment (**a**), nominal 100-μm widths were automatically altered to 70 μm (length of 2 pixels) and 105 μm (length of 3 pixels), whereas pre-adjustment of dimensions to 105-μm grid lines and 385-μm side length of squares resulted in consistent geometries (**b**). Microscopic observation showed differences in consistency between printed pieces without (**c**) and with (**d**) dimensional adjustment.

Improvements to the print quality of MSLA prints were achieved through modifications of pre-processing and post-processing. Slicing is the most important pre-processing step, which can affect fabrication quality [33,34]. Software such as UVtools (github.com/sn4k3/UVtools, accessed on 4 February 2022) can display (Figure 2a,b) pixel illumination units that are not available in most slicing software provided by printer manufacturers. Dimensional uniformities were compromised after automatic slicing (Figure 2a) because the slicer must alter dimensions by rounding lateral lengths to match pixel sizes of the LCD array. These non-uniformities resulted in inconsistent widths of grid lines and shapes of square geometries after printing (Figure 2c). Dimensional modification (to ensure that feature lengths were divisible by the pixel size) resulted in higher consistency in grid widths (Figure 2b) and square shapes (Figure 2d).

Printable files for the Phrozen printer can be prepared with third-party slicing software such as Lychee (used in the present study), allowing for flexible adjustment of the print settings to ensure printing quality. Some resin 3-D printers, such as Form 3 (Formlabs, Somerville, MA, USA), allow for an easy “plug-and-print” strategy to simplify the learning and training processes (formlabs.com/software, accessed on 14 February 2022). However, third-party slicing software cannot be used with these printers, which limit control over the more detailed print settings and prevent customization (unpublished data).

### 3.3. Post-Processing

**Figure 3.**
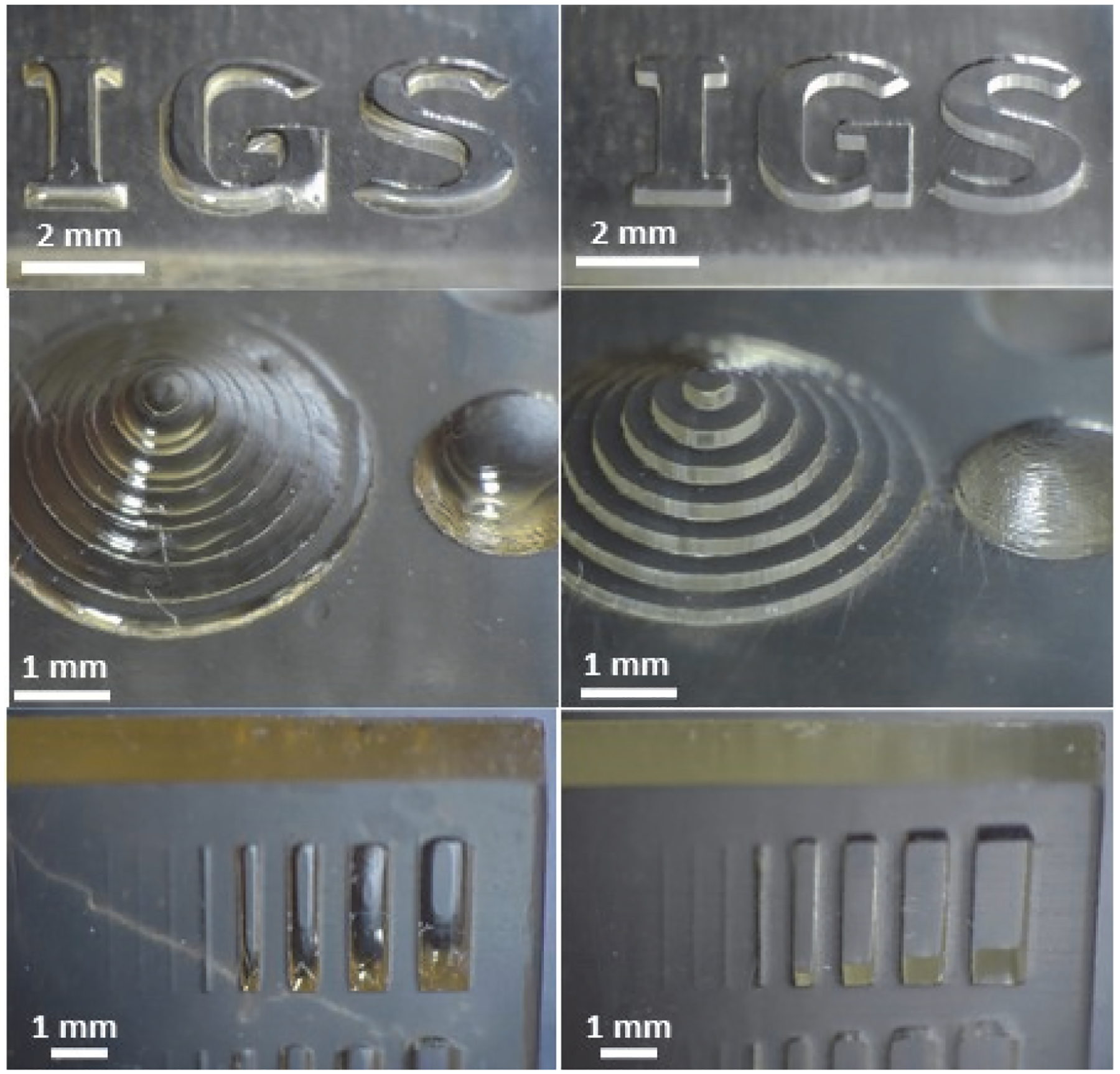
Evaluation of the effects of post-processing on printing quality. Objects were printed with a Phrozen Sonic Mini 4K printer by use of post-processing procedures recommended by the manufacturer (**left panel**) and additional cleaning procedures (**right panel**).

Additional post-processing procedures enhanced printing quality. The standard post-processing method in accordance with the recommendations of the manufacturer resulted in rounding of letters, cylindrical pyramids, sections of spheres, and prisms (Figure 3), indicating that inadequate cleaning led to residual resin that could chamfer edges. Post-processing with improved cleaning (Figure 3) resulted in sharper edges and higher consistency for printing quality in all testing geometries, due to removal of uncured resin that obscured design features.

Although the improved post-processing steps removed substantial uncured resin on print surfaces, it is possible that some uncured resin could remain inside the prints. If objects printed are used as master molds to cast elastomers (e.g., PDMS), various acrylates, monomers, and photoinitiators from uncured resin could inhibit elastomer curing [35]. In these applications, it is critical to perform serial washing, soaking, or surface coating to ensure proper elastomer curing. Different post-processing methods have been developed, including extra washing in solvents [36], extended UV treatment [37], and autoclaving [20]. Additionally, any remaining uncured resin could be cytotoxic to cells [38] and embryos [39,40], but this could be minimized by limiting exposure time. Also, more research is needed to identify the biological effects of different post-processing approaches and outcomes of elastomer curing.

### 3.4. Printer Modification

**Figure 4.**
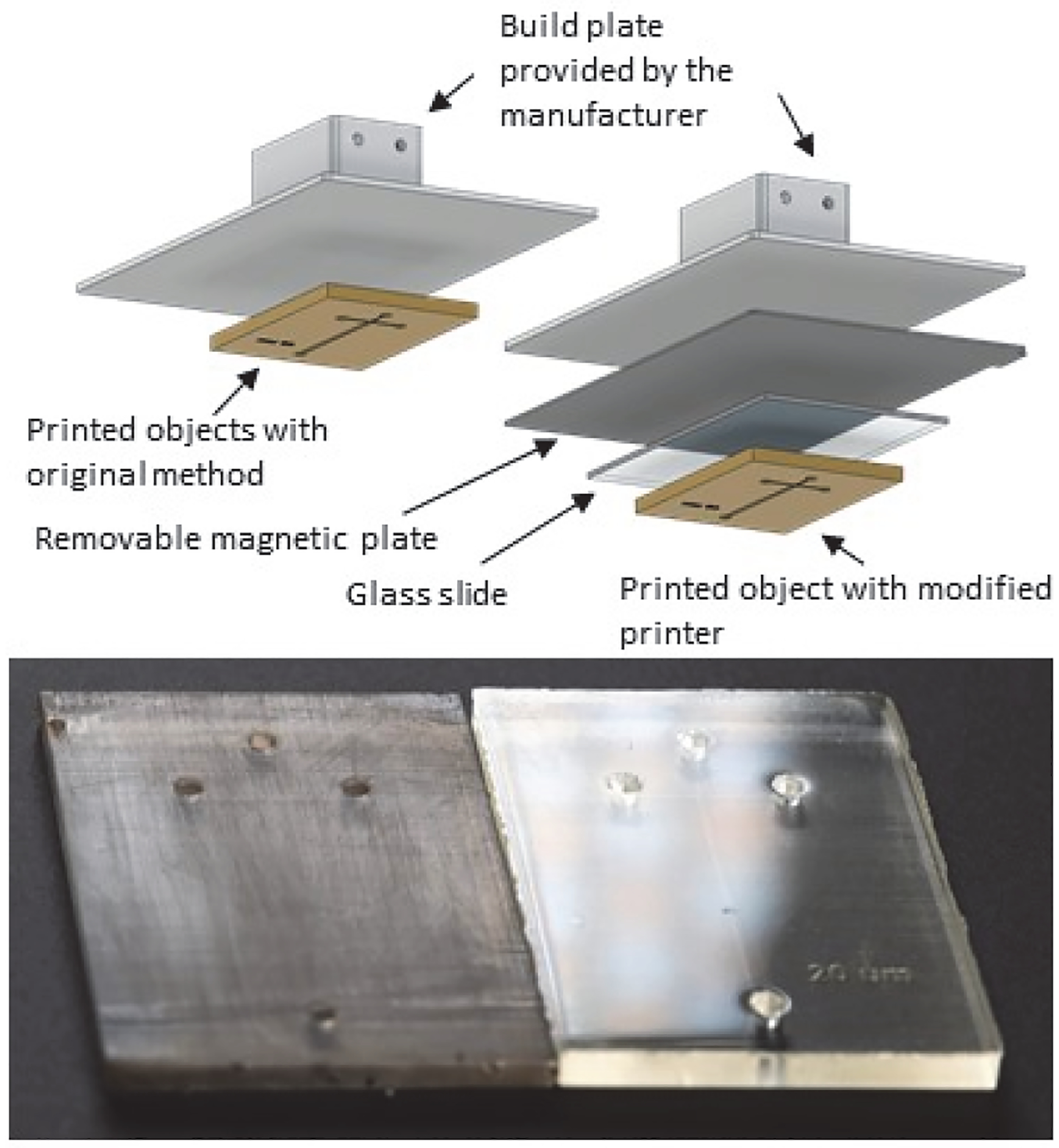
Evaluation of the effects of printer modification on surface smoothness. Addition of a glass slide (5 cm × 7.5 cm) as the modified build surface (upper) for resin 3-D printing can improve the surface smoothness of the base side of the printed objects. The base side of the printed pieces with the original build plate (**bottom left**) showed less smoothness than the piece fabricated with the modified printer (**bottom right**).

The addition of a glass slide on the build plate increased smoothness (Figure 4) of the base (back) side of printed objects. The build plate surfaces of resin printers provided by manufacturers are often treated to form rough surfaces to facilitate print removal. For microfabricated devices that are intended for microscopic observation, however, smooth surfaces are preferred to ensure light transmission and adequate visualization quality. In addition, surface smoothness can allow bonding of two surfaces for complex configurations. The method of printer modification only required super glue and solvents (for glue removal), enabling low-cost application.

### 3.5. Time and Cost Efficiency

**Figure 5.**
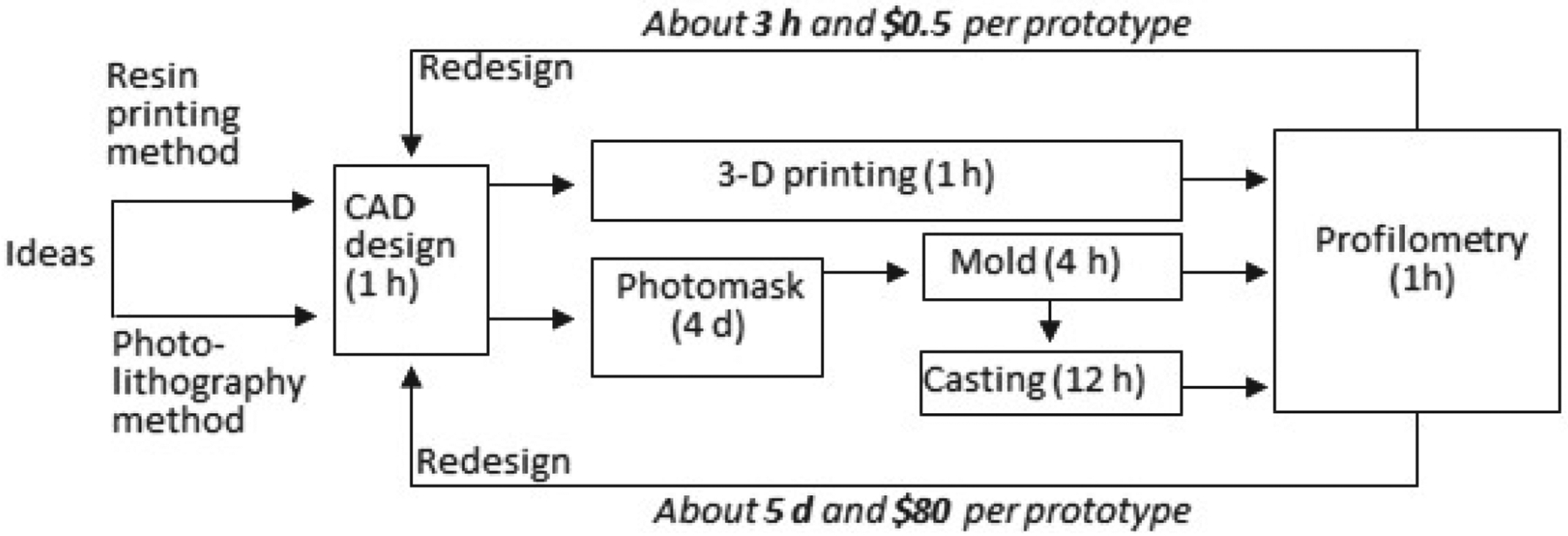
Comparison of estimated costs and time investment to design, manufacture, and evaluate microfabricated prototypes by use of MSLA resin printing and traditional photolithography.

Resin 3-D printing for microfabrication can substantially reduce costs and time compared with photolithography (Figure 5). With the photolithography approach, ideas are converted to designs by CAD software. The design files are often sent to third-party commercial manufacturers to produce high-resolution patterns on photomasks (mask production equipment can cost thousands to hundreds of thousands of dollars, depending on resolution). The photomasks are shipped back, which can take ~4 d from submission of designs. It takes about 16 h to fabricate molds on the silicon wafers and cast PDMS on the molds. While the print time is subject to the printing parameters and size of the prototype, microfluidic devices generally take about 1 h to fabricate with MSLA resin printing (in our experience). In addition, because the printing time is determined by the height of the printed object rather than by its volume, the build plate can be populated with multiple objects with various designs with no increase in printing time.

In this example, it would cost a total of USD 80 to fabricate a batch of prototypes with photolithography, whereas the cost could be reduced to USD 0.5 by use of resin 3-D printing. The total minimal facility investment for photolithography is generally >USD 10,000 (including spin coater, plasma cleaner, vacuum chamber, oven, hotplate, multiple vacuum machines, and chemicals), whereas the total investment for MSLA printing can be <USD 500 (including printer, resin, and post-processing supplies). Although traditional photolithography has a higher cost and fabrication time, it has several advantages, including consistent fabrication quality, higher resolution, reliable mold casting, and easy bonding to other surfaces, and, thus, is well suited for production. Resin 3-D printing offers great potential in rapid testing and prototyping of open hardware [41,42] when applications are not limited by these factors.

## 4. Conclusions

Aquaculture research naturally attracts interdisciplinary collaboration. With the introduction of opportunities for low-cost microfabrication to aquatic biologists, substantial innovation can be anticipated to solve real-world problems in many applications including development of germplasm repositories to support sustainable aquaculture. The wide availability of low-cost resin printing is likely to be highly disruptive in the future because of the reduced bar to entry into the microfabrication field. This explorative work shows that consumer-grade MSLA resin 3-D printers offer great potential in microfabrication, especially for rapid prototyping. The major findings are: (1) fabrication quality of MSLA resin 3-D printing could approach traditional photolithography with features as small as 20 μm and dimensional discrepancy <5%; (2) alterations made to pre-processing, post-processing, and printer configuration steps improved print quality, shown in objects with sharper edges and smoother surfaces; and (3) the prototyping time and cost of resin 3-D printing (3 h with USD 0.5/prototype) were considerably lower than those of traditional photolithography (5 d with USD 80/prototype). Opportunities for low-cost microfabricated devices (e.g., microfluidics, millifluidics, and counting chambers) can quickly expand as printing costs are reduced, the resolution and quality of finished prints are improved, and specialty techniques to implement 3-D printed geometries in microfluidics are developed. Limitations remain in MSLA printing, including the need to replace LCD screens and incomplete light admission through pixels (that could lead to loss of dimensional accuracy). With the rising popularity of low-cost resin printers, advances in LCD technologies will likely address these problems in the near future, and bring in next-generation resolution capabilities (e.g., 8K resolution). Approaches such as these can be especially powerful when presented in the form of open hardware by functioning as force multipliers in addressing large, intractable, global problems, such as ensuring quality management for germplasm repository development in aquatic species [43,44].

## Figures and Tables

**Table 1.** Examples of microfabrication with commercially available resin 3-D printers reported in previous publications and the present work.

Printer	Vendor	Method	Cost (US$)	Resolution (*x*,*y*-Axis, μm) *	Resolution (*z*-Axis, μm) *	Application	Reference
Form 2	Formlabs	Laser SLA	2400	300	150	Cell adhesion and proliferation	[20]
ProJet 7000 HD	3D Systems Inc.	Laser SLA	7200	300	300	Millifluidic channels	[21]
Objet 350	Stratasys Inc.	PolyJet **	5000	300	300	Millifluidic channels	[21]
Objet Eden 260VS	Stratasys Inc.	PolyJet **	19,800	250	250	Microfluidic channels	[22]
Stratasys J750	Stratasys Inc.	PolyJet **	300,000	125	54	Microfluidic channels	[23]
Miicraft+	Miicraft	DLP	5000	250	250	Microfluidic channels	[22]
Flashforge Hunter	Flashforge	DLP	4000	3000	1000	Resin printing toxicity	[24]
Form 3	Formlabs	Laser SLA	3500	25	25	Microelectrode	[25,26]
Phrozen Mini 4K	Phrozen Technology	MSLA	350	35	10	Millifluidic channels	This study

* Highest resolutions provided by manufacturers.

** PolyJet printers dispense photocurable resins in a manner that resembles inkjet printers.

**Table 2.** Printing specifications for resin 3-D printing (Phrozen Mine 4K printer).

Parameters	Specifications
Layer height	10 μm
Bottom layer number	2
Exposure time (bottom layer)	10 s
Light-off delay (bottom layer)	23 s
Exposure time (other layers)	2.8 s
Light-off delay (other layers)	7s
Lifting speed	30 mm/min
